# Use of antibiotics as feed additives: a burning question

**DOI:** 10.3389/fmicb.2014.00334

**Published:** 2014-07-02

**Authors:** Madhab K. Chattopadhyay

**Affiliations:** Centre for Cellular and Molecular Biology (CSIR)Hyderabad, India

**Keywords:** antibiotics, livestock, animal feed, growth-promoting effect, antibiotic-resistance, ban, guidelines for industry

## Introduction

Antibiotics are chemotherapeutic agents used for the clinical management of infectious diseases in humans, plants and animals. However a sizeable fraction of antibiotics produced every year all over the world is used for non-therapeutic purposes. In US alone, about 24.6 million pounds of antibiotics are used in animal agriculture annually and a substantial portion of this is used as growth promoters and not for the treatment of infections (Oliver et al., 2011). According to a recent report, out of 13 million kg of antibiotics administered to animals in 2010, the major portion was meant for promoting the growth of the livestock (Spellberg et al., 2013). The ability of low doses antibiotics to promote growth of animals and birds was discovered serendipitously in the 1940s (Gustafson and Bowen, 1997). Subsequently, it was widely exploited and by this time, addition of antibiotics to the animal feed to stimulate growth has turned into a global practice.

The basis of growth-promoting effect of antibiotics is not clearly known. It is postulated that microorganisms present in the animal feed consume a considerable portion of nutrients in the feed. They also inhibit absorption from the intestine and produce toxins having adverse effect on the health of the animals. The growth-promoting effect of antibiotics might stem from their ability to suppress these harmful organisms. It is also suggested that animals reared in the unhygienic environments always bear some latent infections, which trigger a cascade of events in their immune system. Cytokines produced in the process lead to the release of some catabolic hormones which cause wastage of muscles. Antibiotics relieve the animals of the need to produce cytokines by suppressing the causative agents of infections.

## Benefits associated with the use of antibiotics

Evidences available in the literature speak volumes on the beneficial effects obtained from antibiotics used as a feed additive. Pigs supplemented with antibiotics in their feed require 10–15% less feed to achieve a desired level of growth. Cost of feed constitutes a major portion of the expenses involved in rearing animals. Hence addition of antibiotics substantially cuts down the expenditure. Antibiotics added to the feed also ensure more efficient conversion of feed to animal product and improvement. The daily growth rate of animals subsisting on antibiotic-supplemented food is known to be improved by 1–10% compared to that of the animals provided feed without antibiotic. The meat obtained from antibiotic-fed animals is also of better quality with higher amount of protein and less amount of fat compared to that obtained from animals not supplemented with antibiotics (Hughes and Heritage, 2002). Use of tetracycline and penicillin in chicken feed led to a significant improvement in the production of eggs and hatchability besides feed efficiency (Gustafson and Bowen, 1997). Health of the livestock fed with antibiotic-mixed food is also markedly improved. Following addition of chlortetracycline and sulfamethazine to the feed, the rate of bovine respiratory disease morbidity, the rate of relapses and mortality and also the rate of animals diagnosed with chronic respiratory disease were found to be significantly decreased (Gallo and Berg, 1995). Benefits in terms of the rate and efficiency in the gain of body weight, decrease in mortality and morbidity and reduction in the occurrence of subclinical diseases, were observed using of antibiotics during all phases of growth of pigs (Cromwell, 2002). The adverse effects of inflammation and pro-inflammatory mediators in animals (e.g., reduction in growth, feed intake, reproduction, milk production, and metabolic health) are well-known. The anti-inflammatory potential of antibiotics (particularly macrolides) provides a rational basis of their beneficial effects which is independent of their antimicrobial effect (Buret, 2010). Hence there is no doubt about the important role of antibiotics in profitable and efficient production of livestock.

## Risks associated with the use of antibiotics

On the other hand, use of antibiotics in animal feed as growth-promoters appears to promote emergence of antibiotic-resistant strains. The problem of bacterial resistance to antibiotics is a burning question throughout the world. According to an estimate of the World Health Organization, during the past decade number of deaths caused by some resistant strains exceeded the combined number of deaths caused by influenza, Human Immunodeficiency Virus and traffic accident (Yap, 2013). While emergence of antibiotic-resistant strains is most often correlated with the use of antibiotics, resistance is detected even in bacteria obtained from places which are uninhabited, thinly populated (Chattopadhyay and Grossart, 2010) and totally detached from human intervention (Bhullar et al., 2012). In this backdrop, quite understandably the possibility that presence of antibiotics in the animal feed might contribute to the crisis, has triggered a vigorous controversy. It is widely believed that use of antibiotics as growth promoters promotes evolution and/or selection of antibiotic-resistant strains in animal farms. Reports published from time to time on the isolation of bacterial strains from animals, resistant to the antibiotics that are added to their feed, have fueled the debate further. It is also evident that the possibility of emergence of bacterial strains resistant to the therapeutically useful antibiotics for humans cannot be bypassed by substituting the antibiotics with their analogs in the animal feed. For example avoparcin is a glycopeptide antibiotic not used in humans. Use of this antibiotic as feed additive has been known to be associated with emergence of avoparcin- resistant strains, which are cross-tolerant to vancomycin, a glycopeptide antibiotic used in humans (Marshall and Levy, 2011) Transfer of resistance-conferring genes from the bacteria of animal origin to the bacteria of human origin has also been demonstrated in animal model (Moubareck et al., 2003). Dissemination of resistance is promoted even by sepiolite, a non-antibiotic feed additive, which facilitates horizontal gene transfer in the digestive tract of the animals (Rodríguez-Beltrán et al., 2013).

## The controversy

The proponents for the use of antibiotics in animal feed as growth- promoters however remain unconvinced about the potential of the practice to aggravate the problem of antibiotic resistance (Wallinga and Burch, 2013). They argue that the doses of antibiotics used for this purpose are small compared to their therapeutic doses and it is not definitely known whether such low doses really select for resistance or not. Even those, who accept that agricultural use of antibiotics promotes emergence of antibiotic-resistant strains, believe that evidence of this possibility having a major impact on human health is either non-existent or minimal (Turnidge, 2004). Notwithstanding the fact that bacterial isolates resistant to various antibiotics used in animals are found in humans, it is contended that such people might contract the infection from some other source and it is also possible that both the animals and the humans are infected with the same organism from a common source. Isolates from humans and animals in many cases are claimed to be genetically different (Phillips et al., 2004). The hypothesis on transmission of resistance through food chains is also not universally accepted. Those, who accept it, recommend good hygienic practices in the kitchen and use of vaccines in the birds and animals to reduce the incidence of transmission. Following a ban on the prophylactic use of antibiotics, an overall deterioration of animal health (in terms of diarrhea, weight loss and mortality) was observed in some cases. Hence a ban on the use of antibiotics in animals is believed to be associated with an increased incidence of food-borne diseases in humans as well as more frequent use of antibiotics for therapeutic purposes in animals (Casewell et al., 2003; Spellberg et al., 2013). Therefore restriction on the use of antibiotics as feed additive is considered unwarranted by the proponents. They firmly believe that advantages associated with use of antibiotics in animals outweigh the risks.

On the contrary, it has been demonstrated that exposure to sub-inhibitory concentration of some antibiotics can not only enrich resistant bacteria (Gullberg et al., 2011) but in some cases also stimulate the production of reactive oxygen species which might contribute to an increase in the rate of mutation and emergence of multidrug-resistant mutants (Kohanski et al., 2010). It was also shown that antibiotics in animal feed might facilitate phage-mediated gene transfer thus promoting dissemination of antibiotic resistance (Allen et al., 2011). Horizontal gene transfer, the major mechanism involved in dissemination of antibiotic resistance, is also fostered by sub-inhibitory concentration of some antibiotics (Couce and Blázquez, 2009). The number of food animals exceeds the number of humans by far. Hence use of antibiotics in animal farms poses a risk of creating a large reservoir of resistance genes, the far reaching consequence of which needs hardly to be over-emphasized (Turnidge, 2004). Adverse effects on the health and productivity of animals, observed following a ban on the use of antibiotics in animal feed by the European Union (EU), appeared to be diminished in course of time. Furthermore, the beneficial effects associated with the use of antibiotics were found to be waned in some particular cases (reviewed by Marshall and Levy, 2011). A systematic survey in Danish swine farms indicated improvement in the long-term productivity following decrease in the use of antibiotics in animal feed (Aarestrup et al., 2010). Besides being used as growth-promoters, antibiotics are also widely used for prevention and treatment of the infections of the livestock. Normal microbiota of the organism may be adversely affected by the antibiotics added to the feed. This phenomenon called dysbiosis may foster overgrowth of some already existing harmful microorganisms in the flora (e.g., *Clostridium difficile*), decreased production of short chain fatty acids and other beneficial compounds by the normal flora and increased susceptibility of the livestock to infections (Hawrelak and Myers, 2004). Thus the practice of addition of antibiotics to animal feed might be self-defeating.

## Dilemma faced by the policy makers

Policy makers all over the world are in a quandary to formulate a guideline for the addition of antibiotics to the animal feed. The Guidelines for Industry issued by the Center for Veterinary Medicines of the Food and Drug Administration (FDA, 2012), USA recommend use of antibiotics only for the prevention, control and treatment of infections in animals but not for the promotion of growth, increased performance, and improved feed efficiency. Additionally, use of some antibiotics of critical importance (e.g., the third generation of cephalosporins) is restricted in animal agriculture and they are reserved only for use in humans. Development of suitable alternatives of antibiotics for the clinical management of the infections of the livestock appears to be the need of the hour (Allen et al., 2013). Search for prophylactic measures (e.g., vaccines) for the prevention of diseases is also of crucial importance. Unregulated sale and easy availability of antibiotics have significantly contributed to the problem in many developing countries, where antibiotics are continued to be added to the animal feed as growth promoters. Moreover, unhygienic environment prevailing in the poultries and farm houses in these countries makes the animals more susceptible to infections and necessitates frequent use of antibiotics. Hence it is strongly advocated to do away with or minimize the use of antibiotics by improving the hygiene (Gulland, 2013). It might not be possible to impose a blanket ban (the approach based on precautionary principle adopted by the EU) on the use of antibiotics in animal farms in a global scale. But close monitoring of the situation is imperative everywhere to avert or restrain emergence of resistant strains. The “principle of proof” (gathering evidence before banning a particular compound), adopted by FDA seems to be a practicable approach to contain the conundrum. Recently, FDA has asked the antibiotic-manufacturers to relabel their products voluntarily in order to make people aware of its disapproval for the use of antibiotics as growth-promoters in animals. The label should also indicate that the use of antibiotics in animals must be supervised by a veterinarian. The proposal seeks to put an end to the use of medically useful antibiotics as growth-promoters and also to restrict the scope of prophylactic use of antibiotics in animals against pathogens. However it does not propose for any stricture on the use of non-human antibiotics (e.g., ionophores) in animals as growth-promoters. The initiative is highly appreciated by various organizations and eminent scientists though its success calls for the cooperation of the business lobby (Kuehn, 2014). Use of antibiotics in animal feed remains a highly-debated issue which calls for awareness among common people in the society.

### Conflict of interest statement

The author declares that the research was conducted in the absence of any commercial or financial relationships that could be construed as a potential conflict of interest.
